# Informing patients about their mutation tests: CDKN2A c.256G>A in melanoma as an example

**DOI:** 10.1186/s13053-020-00146-x

**Published:** 2020-07-31

**Authors:** Kari Hemminki, Aayushi Srivastava, Sivaramakrishna Rachakonda, Obul Bandapalli, Eduardo Nagore, Akseli Hemminki, Rajiv Kumar

**Affiliations:** 1 Division of Molecular Genetic Epidemiology, German Cancer Research Center (DKFZ), Im Neuenheimer Feld 580, D-69120 Heidelberg, Germany; 2 Cancer Epidemiology, German Cancer Research Center, D-69120 Heidelberg, Germany; 3 Faculty of Medicine and Biomedical Center in Pilsen, Charles University in Prague, 30605 Pilsen, Czech Republic; 4 Hopp Children’s Cancer Center (KiTZ), Heidelberg, Germany; 5 Division of Pediatric Neurooncology, German Cancer Research Center (DKFZ), German Cancer Consortium (DKTK), Heidelberg, Germany; 6 Division of Functional Genome Analysis (B070), German Cancer Research Center (DKFZ), Heidelberg, Germany; 7 Department of Dermatology, Instituto Valenciano de Oncología, València, Spain; 8 School of Medicine, Universidad Católica de Valencia San Vicente Mártir, València, Spain; 9 Cancer Gene Therapy Group, Translational Immunology Research Program, University of Helsinki, Helsinki, Finland; 10 Comprehensive Cancer Center, Helsinki University Hospital, Helsinki, Finland

**Keywords:** Genetic counseling, Melanoma suppressor gene, Functionality, Deleteriousness

## Abstract

**Background:**

When germline mutations are suspected as causal in cancer, patient DNA may be sequenced to detect variants in relevant genes. If a particular mutation has not been reported in reliable family studies, genetic counselors are facing a dilemma of appropriately informing patients. Many sequencing facilities provide an interpretation of the findings based on the available sequence databases or on prediction tools that are curated from bioinformatics and mechanistic datasets. The counseling dilemma is exacerbated if the pedigree data are not informative but the in silico predictions suggest pathogenicity.

**Methods:**

We present here a real world example of the c.256G > A *CDKN2A* variant, which was detected in one melanoma patient where two siblings were diagnosed with melanoma in situ. We investigated a detailed family history of the affected siblings in order to survey probability of the cancer risks within the context to this mutation.

**Results:**

This c.256G > A *CDKN2A* variant was detected in one of the brothers and in the melanoma-free mother while the other brother in the family tested negative. The variant had been previously described in one patient from a melanoma family. In the family under investigation, the mother’s 16 first-and second-degree relatives had survived past the median onset age for melanoma and none presented melanoma. We tested the variant using multiple bioinformatic tools that all predicted deleteriousness of the variant. The genetic counseling report to the melanoma patient stated that the *CDKN2A* variant was ‘likely pathogenic’ and the disease was defined as ‘likely hereditary melanoma’.

**Conclusions:**

The pedigree data showed at the most a low penetrance variant, which, if taken into consideration, might have altered the provided diagnosis. When dealing with ‘practically’ unknown variants the counselors would be advised to incorporate a detailed family history rather than basing predictions on functionality provided by sequencing facilities.

## Introduction

A study from 2015 concluded: “Clinicians, patients and their relatives would all benefit from an improved level of genetic literacy” [1]. Indeed raising the genetic literacy is a formidable challenge, for cancer alone more than 100 high risk genes are known and many of these are tumor suppressor genes with large coding regions vulnerable to mutations [2, 3]. The core of the literacy problem was exemplified by Richards et al. giving sequencing data on Mendelian disease testing of 5800 persons; 83% of patients had variants that are rare or of uncertain clinical significance (5776 variants) and 17% of patients had pathogenic or “likely pathogenic” variants seen ≥10 times (63 variants) [4]. Several expert groups have provided recommendations for the interpretation of DNA sequence variations in order to help molecular diagnostics, including the American College of Medical Genetics and Genomics and the Association for Molecular Pathology (ACMG/AMP) and the Clinical Genome (ClinGen) [4–6]. We will illustrate the genetic literacy problem with a real world example from a melanoma family with a *CDKN2A* mutation c.256G > A.

**Fig. 1:**
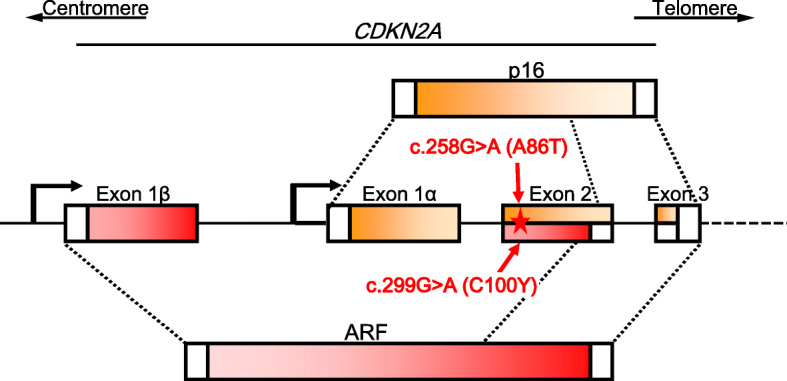
Structure of the CDKN2A locus showing the transcription start sites of proteins p16 and ARF and the exons used. The variant of interest is showen for p16 (on top) and ARF (below)

The most common high-risk predisposing gene for cutaneous melanoma (subsequently melanoma) is *cyclin dependent kinase inhibitor* 2A (*CDKN2A)* [7]. It also predisposes to pancreatic cancer and possibly other cancers [2, 7]. The *CDKN2A* gene encodes two structurally and functionally unrelated proteins, p16^INK4a^ and ARF. P16 and ARF are transcribed from two separate promoters with unique first exons (exon 1α and 1β) and shared exons 2 and 3 that are translated from alternate reading frames and bear no amino acid homology (Fig. 1). P16, a 156 amino acid protein, binds to cyclin dependent kinase 4 and 6 through four ankyrin repeats that results in inhibition of Rb phosphorylation [8, 9]. ARF, a highly basic 132 amino acid peptide, stabilizes p53 through sequestration of MDM2 in nucleolus that inhibits cell cycle in response to oncogenic signals. P16 and ARF have been shown to be markers of senescence in human cells and mouse embryonic fibroblasts, respectively [10]. The a *CDKN2A* mutation c.256G > A causes an Ala86Thr amino acid changed in p16 and Cys100Tyr in ARF where the nucleotide change is c.299G > A. ClinVar database lists 537 variants for *CDKN2A* and of those 43 variants are considered pathogenic with only 6 being missense variants (http://simple-clinvar.broadinstitute.org/). Almost all pathogenic variants were described in syndromes including melanoma. However, germline mutations may also predispose to pancreatic cancer [11].

**Fig. 2:**
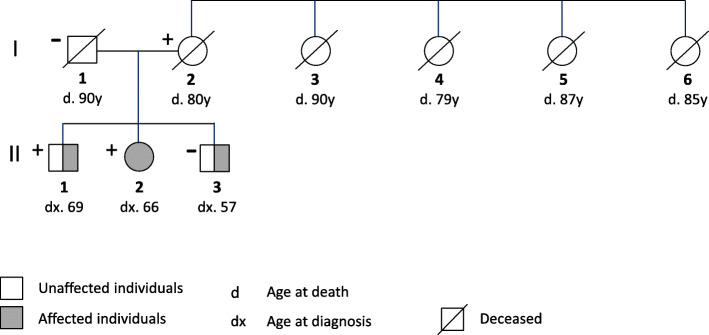
Pedigree of the family of interest. The two generations and each family member labelled. The symbols used are shown below the bedigree

We have been consulting a family in which one sibling was diagnosed with invasive melanoma and two others were diagnosed with in situ melanoma (Fig. 2). *CDKN2A* mutation testing revealed a missense variant c.256G > A in the woman with invasive melanoma (the mutation is indicated in Fig. 1, also giving the resulting amino acid change in ARF). Her first-degree relatives were also tested and her mother and one of the brothers were also positive while her father and younger brother were negative. This mutation has been described so far globally only in one person from a melanoma family [12]. We illustrate the problem of clinical counseling with gene variants that lack data on segregation in cancer pedigrees. We want to point out that taking a family history into consideration may help the counselor to improve his message to the patient.

## Patients and methods

### Family

The family was of a Finnish origin and the members were fair-skinned and blue eyed. The affected female patient was diagnosed with a small invasive melanoma on the leg at the age of 66 years. Her older brother was diagnosed 3 years before her at the age 69 with melanoma on the back, and a younger brother was diagnosed 1 year after her at the age of 57 on the back. Both brothers had melanoma in situ. DNA from expired parents was also available for sequencing. The mothers’ family included four older sisters all of whom survived to an old age. The sisters had altogether 12 children with the current age range from 73 to 88 years; 6 children had died between the ages of 66 to 81 years. As half of the children were alive they were deleted from the pedigree figure. The information about the family history was acquired by a personal interview conducted by a physician, who was also a member of the family.

### Sequencing and counseling

Person II/2 and II/3 were diagnosed with melanoma and in situ melanoma, respectively. As II/1, who lived aboard, was diagnosed earlier with in situ melanoma, the consulting dermatologists referred II/2 and II/3 to medical genetics counseling, where samples were sent for panel sequencing at Blue Genetics. A variant in the *CDKN2A* gene was detected in II/2 but not in II/3. The company classified the c.256G > A as ‘likely pathogenic’ and the consulting medical geneticist described the condition to II/2 as ‘likely hereditary melanoma’ and recommended dermatological check-ups every 6 months and a biannual pancreas cancer screening. An annual dermatological control was recommended for II/3. Information on other risk factors, particularly on solar irradiation was also given, and II/2 was explained that her children may also be gene carriers.

Other family members were sequenced as part of a scientific project using Sanger sequencing for CDKN2A exon 2 specific primers as described previously [13]. Each analysis was repeated twice. The samples from II/2 and II/3 were repeated by Sanger sequencing and the results were consistent with the panel sequencing results.

### In silico annotation

The variant was assessed for conservation and deleteriousness using different prediction tools. High evolutionary conservation suggests functional importance of the position. Genomic Evolutionary Rate Profiling (GERP) [14], PhastCons [15] and PhyloP [16] were used to assess conservation of the variant position. Deleteriousness was predicted using the following tools: CADD v1.5 [17], SIFT [18], PolyPhen V2-HDIV [19], PolyPhen V2-HVAR [19], MutationTaster [20], Mutation Assessor [21], FATHMM [22], PROVEAN [23], VEST3 [24] and RI [25]. The variant was assessed further with SNAP2 [26], a neural network based classifier that distinguishes between effective and neutral variants.

## Results

Mutation analysis for individuals II/2 and II/3 was done by panels sequencing and verified by Sanger sequencing, for other individuals only using Sanger sequencing. The mutational status of the 5 family members is shown in Fig. 2. For all positive individuals the mutation was c.256G > A (Ala86Thr). The mother of the affected siblings died at age of 80 years without melanoma or other known cancer, similar to her 4 sisters. Among the 12 offspring of the 4 sisters none are known to have melanoma but one women died of pancreatic cancer (age 76 years). None of the sisters or their offspring (combined 16 individuals) in generation I or II were known to have melanoma.

**Fig. 3:**
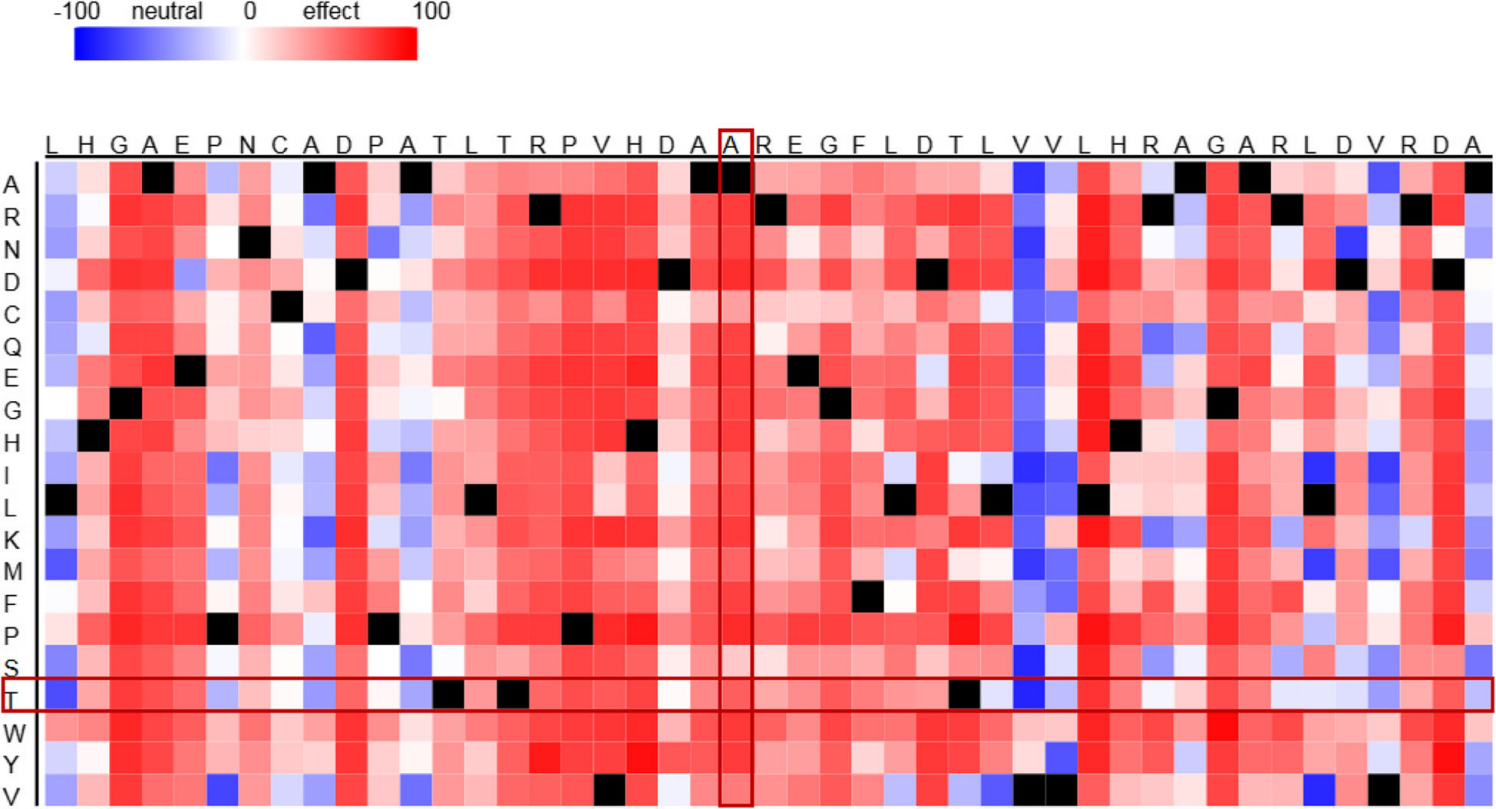
The SNAP2 tool prediction of functionality of the amino acid changes at various locations of the p16 protein approximately centered around Ala86Thr at code 86. The color code illustrates the probability of deleteriousness

SNAP2 predicts functionality of amino acid changes at various locations and in Fig. 3 we show an amino acid segment around Ala86Thr. Almost all changed at amino acid 86 are predicted to be deleterious, as are most other substitutions in this segment. SNAP2 calculates a prediction score for the Ala86The substitution, which is 63 out of a maximum of 100, and the expected accuracy of the prediction is 80%.

We applied a number prediction tools for deleteriousness and conservation for the c.256G > A variant (Table 1). SIFT, PolyPhen, Mutation Taster, PROVEAN and Reliability Index uniformly predict deleteriousness. CADD score of 33 is high. The locus is highly conserved.

**Table 1:** Deleteriousness and conservation scores for the CDKN2A variant c.256G > A

Tools	Score	Interpretation
Deleteriousness Scores		
**SIFT**	Deleterious (D)	D – Damaging/Deleterious (> 0.05) T – Tolerated (< 0.05)
**PolyPhen-v2**	PolyPhen2_HDIV = 1.000 (D) PolyPhen2_HVAR = 0.945 (D)	D – Probably damaging (> 0.908) P – Possibly damaging (0.446 < score ≤ 0.908) B – Benign (≤ 0.446)
**MutationTaster**	0.957 (D)	A – Disease causing automatic D – Disease causing (> 0.5) N – Polymorphism (< 0.5) P – Polymorphism automatic
**MutationAssessor**	No data	–
**FATHMM**	No data	–
**VEST3**	No data	–
**PROVEAN**	−3.711 (D)	D – Damaging/Deleterious (≤ −2.5) N – Neutral (> − 2.5)
**Reliability Index (RI)**	6	D – Damaging (≥ 5) N – Neutral (< 5)
**CADD v1.5**	33	CADD > 10 = In top 10%, CADD > 20 = In top 1%, CADD > 30 = In top 0.1% of probable deleterious variants in the human genome
Conservation Scores		
**GerpN**	5.93	Highly conserved (> 2)
**PhastCons**	0.999	Highly conserved (> 0.3)
**PhyloP**	5.697	Highly conserved (> 3.0)

## Discussion

High-risk *CDKN2A* mutations significantly contribute to the genetic architecture of melanoma as these are found in about 30% of patients in families of three or more affected individuals [27]. According to a Swedish study, *CDKN2A* positive families accounted for 11.5% (31/269) of all melanoma families, and the positivity correlated with the number of affected individuals; in the positive families a median of 6 individuals melanomas were diagnosed compared to 2 individuals in mutation negative families [28]. However, we have earlier shown that in the Swedish population families of 3 or more diagnosed melanoma cases accounted for less than 8% of familial melanoma [29]. Thus the present sibship of 3 affected cases is rare; however, it may be noted that the brothers had only melanoma in situ. Family studies have shown that the melanoma in situ show approximately an equal familial risk of about 2.5–3.0 as does invasive melanoma [30, 31].

It is known that life-time risk (penetrance) of melanoma in carriers of CDKN2A mutations may be modified by other genetic and environmental factors [7]. Solar radiation is a likely environmental factor which was invoked as an explanation why penetrance of CDKN2A mutations in high-risk families is higher in Australia than in Europe [27]. However, a later study did not find difference in penetrance and considered the contribution of ambient solar radiation unlikely [32]. The population-based penetrance of CDKN2A mutations has been estimated to be 14% by age 50 years and 28% by age 80 years based on Australian and North American populations [33].

The detected mutation caused a change in two proteins, p16 and ARF, and it is not possible to assign the effects, if any, to one or the other, even though much of the global literature concerns p16. Our in silico analysis of the detected variant using the various prediction tools suggested overwhelmingly genetic consequences, but we do not know how the ‘likely pathogenic’ classification by Blue Genetics was derived at. This is, however, contradicted by the overwhelming pedigree data. Firstly, one of the affected brothers was negative and the mother with mutation was melanoma-free until her death at age 80 years. Secondly, the mother’s 4 sisters and their 12 offspring were melanoma-free into an advanced age, well past the median age of melanoma diagnosis (64 years) [34]. As it can be assumed that 50% of the 4 sisters and 25% of their offspring were mutation carriers the penetrance of the mutation is low. Of course, de novo mutation cannot be excluded. However one person was diagnosed with pancreatic cancer but at a relatively advanced age of 76 years. Factors other than *CDKN2A* mutations may have contributed to melanoma risk. Family members share skin types and many habits. The affected 3 siblings were fair-skinned and were not protected against sun bathing in their youth. They reported several sun burning episodes during early summers.

In summary, without pedigree data it is justified to consider CDKN2A c.256G > A mutations likely pathogenic. However, the available pedigree data in this case suggest that the penetrance is low but that does not entirely exclude a low level of pathogenicity. To avoid unnecessary fear, genetic counseling should collect a detailed family history, and use such information to modify the advice to patients and their relatives. All patients have families and the majority would be highly motivated to collaborate with the counsellors. As this case demonstrates, a genetic change alone is not sufficient to tell the proband or family members how high the risk of melanoma(s) is in their family or even mutation carriers. A family history, and perhaps an investigation into behavior, such as sunbathing in this case, would help the geneticist to assess the penetrance of the mutation found. In essence, the penetration of a mutation is the combined outcome of genetic modifying factors together with behavioral factors.

### Ethical statement

Genetic testing was approved by the ethical board of the Instituto Valenciano de Oncologia, Valencia, Spain. Two of the patients underwent clinical genetic counseling at the University Hospital in Tampere, Finland. The living individuals gave an informed consent for mutation testing.

## Data Availability

Panel sequencing data are not available because it was ordered by the hospital from a private sequencing company. All other data are available upon request.

## References

[1] Eccles DM, Mitchell G, Monteiro AN, Schmutzler R, Couch FJ, Spurdle AB (2015). BRCA1 and BRCA2 genetic testing-pitfalls and recommendations for managing variants of uncertain clinical significance. Ann Oncol.

[2] Rahman N (2014). Realizing the promise of cancer predisposition genes. Nature..

[3] Vogelstein B, Papadopoulos N, Velculescu VE, Zhou S, Diaz LA, Kinzler KW (2013). Cancer genome landscapes. Science..

[4] Richards S, Aziz N, Bale S, Bick D, Das S, Gastier-Foster J (2015). Standards and guidelines for the interpretation of sequence variants: a joint consensus recommendation of the American College of Medical Genetics and Genomics and the Association for Molecular Pathology. Genet Med.

[5] Rehm HL, Berg JS, Brooks LD, Bustamante CD, Evans JP, Landrum MJ (2015). ClinGen--the clinical genome resource. N Engl J Med.

[6] Li MM, Datto M, Duncavage EJ, Kulkarni S, Lindeman NI, Roy S (2017). Standards and guidelines for the interpretation and reporting of sequence variants in Cancer: a joint consensus recommendation of the Association for Molecular Pathology, American Society of Clinical Oncology, and College of American Pathologists. J Mol Diagn.

[7] Read J, Wadt KA, Hayward NK (2016). Melanoma genetics. J Med Genet.

[8] Kumar R, Sauroja I, Punnonen K, Jansen C, Hemminki K (1998). Selective deletion of exon 1 beta of the p19ARF gene in metastatic melanoma cell lines. Genes Chromosomes Cancer..

[9] Kumar R, Smeds J, Lundh Rozell B, Hemminki K (1999). Loss of heterozygosity at chromosome 9p21 (INK4-p14ARF locus): homozygous deletions and mutations in the p16 and p14ARF genes in sporadic primary melanomas. Melanoma Res.

[10] Sharpless NE, Sherr CJ (2015). Forging a signature of in vivo senescence. Nat Rev Cancer.

[11] Petersen GM (2016). Familial pancreatic cancer. Semin Oncol.

[12] Bruno W, Ghiorzo P, Battistuzzi L, Ascierto PA, Barile M, Gargiulo S (2009). Clinical genetic testing for familial melanoma in Italy: a cooperative study. J Am Acad Dermatol.

[13] Gast A, Scherer D, Chen B, Bloethner S, Melchert S, Sucker A (2010). Somatic alterations in the melanoma genome: a high-resolution array-based comparative genomic hybridization study. Genes Chromosomes Cancer.

[14] Cooper GM, Stone EA, Asimenos G, Program NCS, Green ED, Batzoglou S (2005). Distribution and intensity of constraint in mammalian genomic sequence. Genome Res.

[15] Siepel A, Bejerano G, Pedersen JS, Hinrichs AS, Hou M, Rosenbloom K (2005). Evolutionarily conserved elements in vertebrate, insect, worm, and yeast genomes. Genome Res.

[16] Pollard KS, Hubisz MJ, Rosenbloom KR, Siepel A (2010). Detection of nonneutral substitution rates on mammalian phylogenies. Genome Res.

[17] Kircher M, Witten DM, Jain P, O'Roak BJ, Cooper GM, Shendure J (2014). A general framework for estimating the relative pathogenicity of human genetic variants. Nat Genet.

[18] Kumar P, Henikoff S, Ng PC (2009). Predicting the effects of coding non-synonymous variants on protein function using the SIFT algorithm. Nat Protoc.

[19] Adzhubei I, Jordan DM, Sunyaev SR (2013). Predicting functional effect of human missense mutations using PolyPhen-2. Curr Protoc Hum Genet.

[20] Schwarz JM, Cooper DN, Schuelke M, Seelow D (2014). MutationTaster2: mutation prediction for the deep-sequencing age. Nat Methods.

[21] Reva B, Antipin Y, Sander C (2011). Predicting the functional impact of protein mutations: application to cancer genomics. Nucleic Acids Res.

[22] Shihab HA, Gough J, Cooper DN, Day IN, Gaunt TR (2013). Predicting the functional consequences of cancer-associated amino acid substitutions. Bioinformatics..

[23] Choi Y, Sims GE, Murphy S, Miller JR, Chan AP (2012). Predicting the functional effect of amino acid substitutions and Indels. PLoS One.

[24] Carter H, Douville C, Stenson PD, Cooper DN, Karchin R (2013). Identifying Mendelian disease genes with the variant effect scoring tool. BMC Genomics.

[25] López-Ferrando V, Gazzo A, de la Cruz X, Orozco M, Gelpí JL (2017). PMut: a web-based tool for the annotation of pathological variants on proteins, 2017 update. Nucleic Acids Res.

[26] Hecht M, Bromberg Y, Rost B (2015). Better prediction of functional effects for sequence variants. BMC Genomics.

[27] Goldstein AM, Chan M, Harland M, Hayward NK, Demenais F, Bishop DT (2007). Features associated with germline CDKN2A mutations: a GenoMEL study of melanoma-prone families from three continents. J Med Genet.

[28] Helgadottir H, Hoiom V, Tuominen R, Nielsen K, Jonsson G, Olsson H, et al. Germline CDKN2A Mutation Status and Survival in Familial Melanoma Cases. J Natl Cancer Inst. 2016;108(11).. 10.1093/jnci/djw135 27287845

[29] Frank C, Sundquist J, Hemminki A, Hemminki K (2017). Risk of other cancers in families with melanoma: novel familial links. Sci Rep.

[30] Chen T, Hemminki K, Kharazmi E, Ji J, Sundquist K, Fallah M (2014). Multiple primary (even in situ) melanomas in a patient pose significant risk to family members. Eur J Cancer.

[31] Hemminki K, Zhang H, Czene K (2003). Familial and attributable risks in cutaneous melanoma:effects of proband and age. J Invest Dermatol.

[32] Cust AE, Harland M, Makalic E, Schmidt D, Dowty JG, Aitken JF (2011). Melanoma risk for CDKN2A mutation carriers who are relatives of population-based case carriers in Australia and the UK. J Med Genet.

[33] Begg CB, Orlow I, Hummer AJ, Armstrong BK, Kricker A, Marrett LD (2005). Lifetime risk of melanoma in CDKN2A mutation carriers in a population-based sample. J Natl Cancer Inst.

[34] Robsahm TE, Helsing P, Nilssen Y, Vos L, Rizvi SMH, Akslen LA (2018). High mortality due to cutaneous melanoma in Norway: a study of prognostic factors in a nationwide cancer registry. Clin Epidemiol.

